# Variant adiponutrin confers genetic protection against cholestatic itch

**DOI:** 10.1038/srep06374

**Published:** 2014-10-09

**Authors:** Marcin Krawczyk, Malgorzata Milkiewicz, Hanns-Ulrich Marschall, Clemens Bartz, Frank Grünhage, Ewa Wunsch, Piotr Milkiewicz, Frank Lammert

**Affiliations:** 1 Department of Medicine II, Saarland University Medical Center, Saarland University, Homburg, Germany; 2 Medical Biology Laboratory, Pomeranian Medical University, Szczecin, Poland; 3 Sahlgrenska Academy, Institute of Medicine, Department of Molecular and Clinical Medicine, University of Gothenburg, Gothenburg, Sweden; 4 Department of Gynecology and Obstetrics, Klinikum Saarbrücken, Saarbrücken, Germany; 5 Liver Research Laboratories, Pomeranian Medical University, Szczecin, Poland; 6 Liver and Internal Medicine Unit, Department of General, Transplant and Liver Surgery, Medical University of Warsaw, Warsaw, Poland; 7 These authors contributed equally to this work.

## Abstract

Lysophosphatidic acid (LPA) mediates cholestatic pruritus. Recently the enzyme PNPLA3, expressed in liver and skin, was demonstrated to metabolise LPA. Here we assess the association of the *PNPLA3* variant p.Ile148Met, known to be associated with (non-)alcoholic fatty liver disease (NAFLD) in genome-wide association studies, with cholestatic itch in 187 patients with primary biliary cirrhosis (PBC) and 250 PBC-free controls as well as 201 women with intrahepatic cholestasis of pregnancy (ICP) and 198 female controls without a history of ICP. Our hypothesis was that the intensity of cholestatic itch differs in carriers of distinct *PNPLA3* p.Ile148Met genotypes. Patients with PBC carrying the allele p.148Met that confers an increased NAFLD risk reported less itching than carriers of the p.148Ile allele (ANOVA P = 0.048). The *PNPLA3* p.148Ile allele increased the odds of requiring plasmapheresis for refractory pruritus (OR = 3.94, 95% CI = 0.91–17.00, P = 0.048). In line with these findings, the *PNPLA3* p.148Met allele was underrepresented in the ICP cohort (OR = 0.66, 95% CI = 0.47–0.92, P = 0.013). Notwithstanding the need for further replication of these findings, we conclude that the *PNPLA3* allele p.148Met might confer protection against cholestatic pruritus, possibly due to increased LPA-acyltransferase activity in liver and/or skin.

Recent studies established the adiponutrin (*PNPLA3*) variant p.Ile148Met as common genetic risk factor for severe forms of chronic liver diseases^1^. Indeed, patients with non-alcoholic^2^,^3^ and alcoholic fatty liver disease^4^,^5^ who carry the p.148Met allele at this locus are prone to progressive liver fibrosis and cirrhosis. In our elastography-based analysis^6^, we demonstrated that this allele is associated with increased liver fibrosis in patients with chronic liver diseases in general. PNPLA3 has been reported to be a triacylglycerol hydrolase or lysophosphatidic acid (LPA) acyltransferase, and the p.Ile148Met variant might affect the remodelling of lipids, including the conversion of LPA into phosphatidic acid^1^,^7^. Carriers of the p.148Met allele might have have increased LPA catabolism, which promotes the synthesis of diacylglycerol and modulates the composition of lipid droplets.

To date, the treatment of pruritus in patients with liver diseases remains a troublesome challenge. Although several drugs (ursodeoxycholic acid, rifampicin) and invasive approaches (nasobiliary drainage, plasmapheresis) represent therapeutic options^8^, they are not effective in many patients. Clinical observations support the notion that patients with comparable grade of cholestasis display different severity of pruritus^9^. Thus genetic predisposition might affect the degree of itching, but to date no common pruritus-related genes that are associated with treatment-refractory pruritus have been identified. In addition to liver, skin also shows high *PNPLA3* expression levels^10^. Interestingly, LPA has been identified as the critical mediator of cholestatic pruritus^11^. Indeed, LPA binds to the LPA_1_ receptor in skin and causes itching^11^. Patients with cholestatic pruritus are characterized by increased serum concentrations of LPA, which can be generated from lysosphatidylcholine by the enzyme autotoxin in blood^11^. Therefore we hypothesized that the polymorphism p.Ile148Met of *PNPLA3*, which catabolizes LPA, affects the severity of cholestatic pruritus and investigated two independent cohorts of patients with cholestatic liver diseases (Tables 1 and 2).

## Results

**Figure 1:**
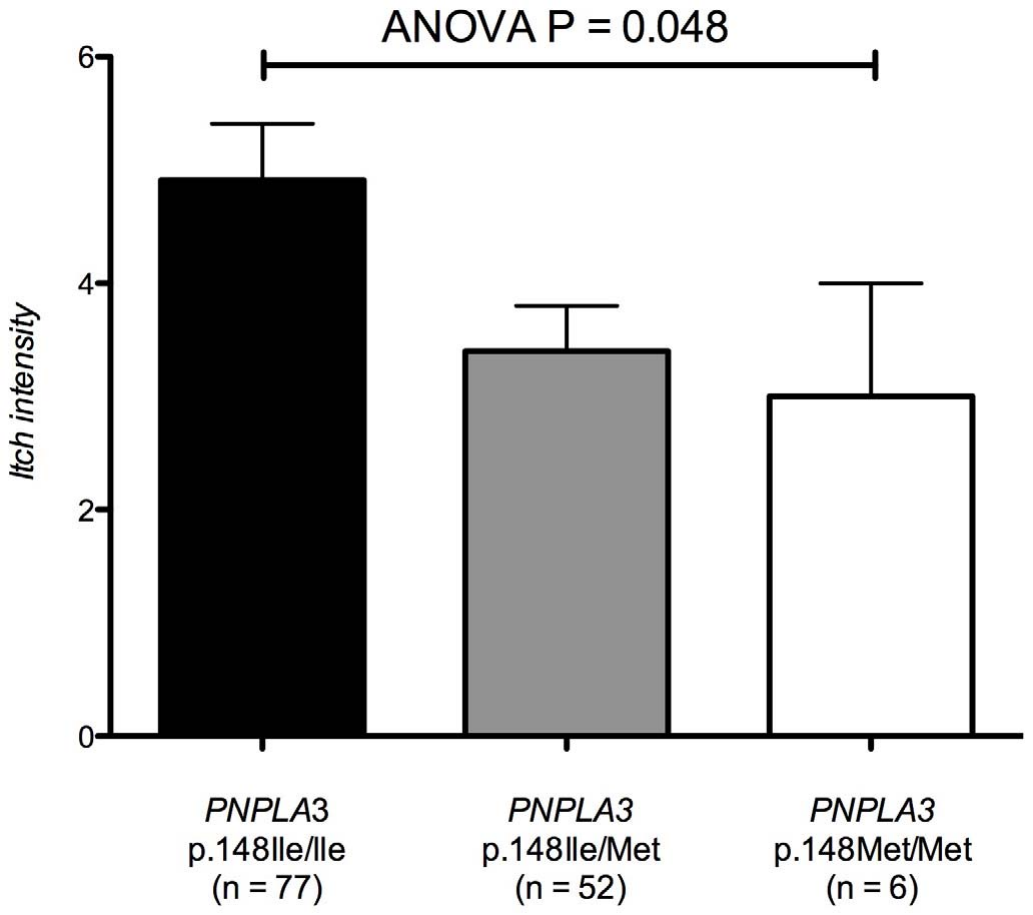
Itch intensity in PBC patients stratified for *PNPLA3* p.Ile148Met genotypes. Carriers of different *PNPLA3* p.Ile148Met genotypes report significantly different intensity of pruritus (ANOVA P = 0.048), as quantified by the itch domain of the PBC-40 questionnaire.

**Figure 2:**
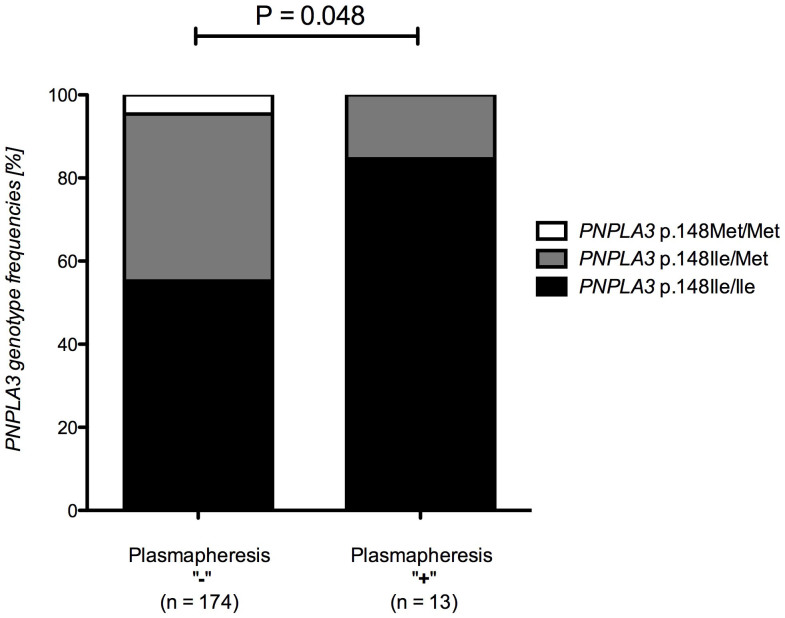
*PNPLA3* p.Ile148Met genotypes in PBC patients who did not and patients who required plasmapheresis for the treatment of pruritus. Patients who were treated with plasmapheresis carry the *PNPLA3* allele p.148Ile significantly (allelic 1-df test P = 0.048) more often than patients who did not require this treatment.

As shown in Figure 1 and Supplementary Table 1, the intensity of itch differed significantly between carriers of different genotypes of the *PNPLA3* variant p.Ile148Met (ANOVA P = 0.048). Moreover, the *PNPLA3* allele p.148Ile conferred a significant risk (OR = 3.94, 95% CI = 0.91–17.00, P = 0.048) to require plasmapheresis (Figure 2, Table 3). On the other hand, this variant did not increase the risk of developing PBC *per se* (Supplementary Table 2), or cirrhosis in PBC patients (Supplementary Table 3), and was neither associated with laboratory parameters of PBC (Supplementary Table 4) nor with domains of the PBC-40 questionnaires other than the intensity of pruritus (Supplementary Table 1).

**Figure 3:**
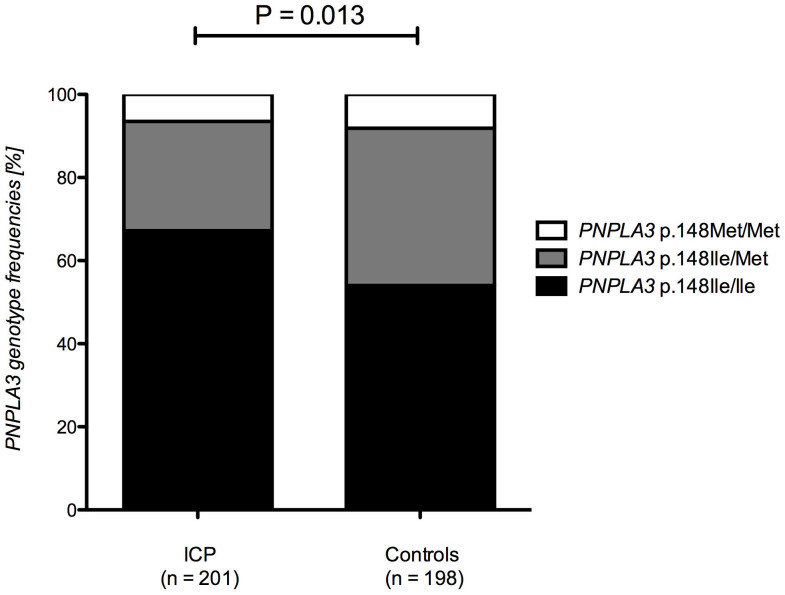
*PNPLA3* p.Ile148Met genotypes in ICP patients and sex-matched controls. Patients diagnosed with ICP present a significantly (allelic 1-df test P = 0.013) higher frequency of the *PNPLA3* allele p.148Ile as compared to controls.

Consistent with these observations, we detected a significantly increased frequency of the *PNPLA3* allele p.148Ile in a second large independent cohort of patients with ICP who presented with itch during pregnancy as compared to controls (Figure 3, Supplementary Table 5). As illustrated in Supplementary Figure 1, this lead to a departure of the genotype distribution from Hardy-Weinberg equilibrium in ICP patients, supporting the genetic association. Patients carrying the *PNPLA3* allele p.148Met were at significantly decreased risk (OR = 0.66, 95% CI = 0.47–0.92, P = 0.013) of presenting with ICP. Since pruritus is the major symptom at diagnosis of ICP, the lower frequency of the p.148Met allele in this cohort replicates the observation that this allele decreases the degree of cholestatic itch.

## Discussion

This is the first study demonstrating an association of a genetic polymorphism with cholestatic pruritus. Here we report that the *PNPLA3* allele p.148Met decreases itch severity in cholestatic patients. Our results are in line with functional analyses implicating that carriers of this allele display an increased metabolism of LPA^7^. We hypothesize that increased LPAAT^7^ activities in liver and/or skin^10^ of patients carrying the *PNPLA3* allele p.148Met might be the mechanism that decreases itching, however further functional analyses are required. Moreover, our study identifies this *PNPLA3* variant as potential genetic marker for therapy-refractory pruritus. Interestingly, our recently reported young female patient who developed severe refractory pruritus after acute hepatitis A infection as a result of hepatobiliary transporter variants^12^ is also a homozygous carrier of the susceptible *PNPLA3* allele p.148Ile (M.K. and F.L., data on file).

The association between the *PNPLA3* polymorphism and pruritus was independent from potential effects on liver function^2^,^3^,^4^,^5^,^6^,^13^ (Supplementary Tables 3 and 4). The lack of association of the *PNPLA3* variant with liver cirrhosis in the PBC cohort indicates that the skin might be the major site where the antipruritic effects are exerted. Hence we conclude that the *PNPLA3* variant p.Ile148Met is an example of biological pleiotropy^14^,^15^ with influence on more than one liver-related trait: In patients with chronic liver diseases the allele p.148Met is associated with disease progression^16^, but in case of cholestasis it might protect against itch. However, our results require further replication in additional cohorts. Since the *PNPLA3* allele p.148Ile may to a certain extent explain the development of therapy-refractory pruritus, genotyping of the *PNPLA3* variant p.Ile148Met might be included in the diagnostic work-up of patients with cholestatic liver conditions.

## Patients and Methods

### Patients with primary biliary cirrhosis (PBC)

In total, we recruited 187 Polish PBC patients (age range 22–83 years, 166 females). All patients fulfilled the European Association for the Study of the Liver (EASL) criteria for the diagnosis of PBC^8^. Table 1 presents the detailed description of this cohort. Liver function tests were determined by standard assays in fasted blood samples. In 135 patients, quality of life and intensity of itch were prospectively assessed with the PBC-40 questionnaire^17^. Sixty-nine patients presented with histological, clinical and/or imaging features characteristic for liver cirrhosis. Among the PBC patients, a total of 13 who did not respond to pharmacological treatment of their pruritus (including ursodeoxycholic acid, colestyramine and rifampicin) were treated with plasmapheresis. The control cohort encompassed 250 healthy blood donors from the National Blood Services (Table 1)^18^.

### Patients with intrahepatic cholestasis of pregnancy (ICP)

A cohort of 201 females with ICP (age range 17–46 years) was recruited between 2000 and 2013. Table 2 summarizes the details of this cohort. The included patients fulfilled the EASL criteria for the diagnosis of ICP^8^. The control cohort consisted of 198 non-pregnant females (age range 20–60 years) without any documented episodes of cholestasis during pregnancy. The study was conducted according to a study design approved by the local ethical committees, and informed consent was obtained from all study participants.

### Genotyping

In all individuals, we genotyped the *PNPLA3* variant p.Ile148Met (rs738409) as described^6^. Genomic DNA was isolated from EDTA anticoagulated blood using the membrane-based QIAamp DNA extraction protocol (Qiagen, Hilden, Germany).

### Statistical analyses

The consistency of genotype frequencies with Hardy-Weinberg equilibrium (HWE) was tested using an exact test. Allele frequency differences were assessed by 1-df chi^2^ tests (http://ihg.gsf.de/cgi-bin/hw/hwa1.pl). The study hypothesis was tested at a two-sided p-value of 0.05. For further exploratory analyses, quantitative phenotypic data were analyzed using Mann-Whitney U or Fisher's protected least significant difference (PLSD) tests, and qualitative phenotypes were assessed in contingency tables.

## Author Contributions

M.K., E.W., H.U.M., C.B., F.G., P.M. and F.L. recruited patients and controls for this study; M.M. performed the genotyping; M.K., M.M., P.M. and F.L. analyzed the data; M.K., P.M. and F.L. drafted and edited the manuscript; P.M. and F.L. supervised the project and contributed equally to this study. M.K. had full access to all the data in the study and takes responsibility for the integrity of the data and the accuracy of the data analysis.

## Supplementary Material

Supplementary Information Supplementary Dataset 1

## Figures and Tables

**Table 1:** Demographic, clinical and laboratory data in PBC patients and controls

	PBC (n = 187)	Controls (n = 250)
**Age (years)**	56 (22–83)	25 (18–66)
**Gender (women/men)**	166/21	210/40
**Liver cirrhosis (yes/no/unknown)**	69/115/3	0/250/0
**AMA (positive/negative)**	162/25	ND
**ALT (IU/l)**	85 (10–987)	ND
**AP (IU/l)**	323 (37–1899)	ND
**γ-GT (IU/l)**	295 (11–1932)	ND
**Bilirubin (mg/dl)**	3.2 (0.2–45.5)	ND

Values are given as medians (ranges), unless stated otherwise.

Abbreviations: ALT, alanine aminotransferase; AMA antimitochondrial antibodies; AP, alkaline phosphatase; γ-GT, γ-glutamyl transpeptidase; ND, not done; PBC, primary biliary cirrhosis.

**Table 2:** Demographic, clinical and laboratory data in ICP patients and controls

	ICP (n = 201)	Controls (n = 198)
**Age (years)**	30 (17–46)	49 (20–60)
**Gender (women/men)**	201/0	198/0
**Liver cirrhosis (yes/no)**	0/201	0/198
**ALT (IU/l)**	112 (4–1196)	38 (11–194)
**AP (IU/l)**	312 (58–1829)	ND
**γ-GT (IU/l)**	30 (5–473)	34 (12–1138)
**Bilirubin (mg/dl)**	0.7 (0.2–15.0)	0.5 (0.2–1.3)

Values are given as medians (ranges), unless stated otherwise.

Abbreviations: see Table 1A. ICP, intrahepatic cholestasis of pregnancy.

**Table 3:** Distribution of *PNPLA3* alleles and genotypes in PBC patients stratified according to the need for plasmapheresis to relieve pruritus

	Count of alleles/genotypes	
*PNPLA3* p.Ile148Met alleles/genotypes	Plasmapheresis (+) (n = 13)	Plasmapheresis (−) (n = 174)
Ile	24 (92.3)	262 (76.3)
Met	2 (7.7)	86 (24.7)
Ile/Ile	11 (84.6)	96 (55.2)
Ile/Met	2 (15.4)	70 (40.2)
Met/Met	0 (0)	8 (4.6)
**Allelic 1-df test**	**P**	**OR (95% CI)**
[Ile] ↔ [Met]	0.048	3.94 (0.91–17.00)

Abbreviations: CI, confidence interval; Ile, isoleucine; Met, methionine; OR, odds ratio; p, protein (amino acid number); PBC, primary biliary cirrhosis; *PNPLA3*, adiponutrin.
