# Supplementary material for: Variant adiponutrin confers genetic protection against cholestatic itch
Source: Sci Rep. 2014 Oct 9;4:6374. doi: 10.1038/srep06374 (PMC4190538; doi:10.1038/srep06374)
Supplement: Supplementary Information — Supplementary Dataset 1 [file srep06374-s1.docx]

**Supplementary Material**

**Variant adiponutrin confers genetic protection against cholestatic itch**

Marcin Krawczyk, Ewa Wunsch, Hanns-Ulrich Marschall, Clemens Bartz, Frank Grünhage, Malgorzata Milkiewicz, Piotr Milkiewicz and Frank Lammert

**Supplementary Table 1**

*PNPLA3* genotypes and domains of PBC-40 in PBC patients

|  | ***PNPLA3* p.Ile148Met variant** | |  |
| --- | --- | --- | --- |
| **Variables** | **[Ile/Ile]** | **[Ile/Met] + [Met/Met]** | **P** |
| **Itch** | 4.9 ± 0.5 | 3.4 ± 0.4 | 0.03 |
| **Fatigue** | 28.2 ± 1.2 | 28.1 ± 1.4 | 0.95 |
| **Cognitive** | 13.7 ± 0.6 | 13.0 ± 0.7 | 0.48 |
| **Social and emotional** | 28.3 ± 1.1 | 30.9 ± 1.4 | 0.16 |
| **Other symptoms** | 16.9 ± 0.6 | 16.5 ± 0.7 | 0.67 |

Abbreviations: Ile, isoleucine; Met, methionine; *PNPLA3*, adiponutrin.

**Supplementary Table 2**

Distribution of *PNPLA3* alleles and genotypes in PBC patients and controls

|  | **Count of alleles / genotypes** | |
| --- | --- | --- |
| ***PNPLA3* p.Ile148Met alleles / genotypes** | **PBC (%)  (n = 187)** | **Controls (%) (n = 250)** |
| Ile | 285 (76.2) | 382 (76.4) |
| Met | 89 (23.8) | 118 (23.6) |
| Ile/Ile | 106 (56.7) | 151 (60.4) |
| Ile/Met | 73 (39.0) | 80 (32.0) |
| Met/Met | 8 (4.3) | 19 (7.6) |
| **Allelic 1-df test** [Ile] ↔ [Met] | **P** 0.946 | **OR (95% CI)** 0.99 (0.72 – 1.35) |

Abbreviations: CI, confidence interval; Ile, isoleucine; Met, methionine; OR, odds ratio; p, protein (amino acid number); PBC, primary biliary cirrhosis; *PNPLA3*, adiponutrin.

**Supplementary Table 3**

Distribution of *PNPLA3* alleles and genotypes in PBC patients

with and without cirrhosis

|  | **Count of alleles / genotypes** | |
| --- | --- | --- |
| ***PNPLA3* p.Ile148Met alleles / genotypes** | **Cirrhosis (%)  (n = 69)** | **No cirrhosis (%) (n =115)** |
| Ile | 100 (72.4) | 182 (79.1) |
| Met | 38 (27.6) | 48 (20.9) |
| Ile/Ile | 34 (49.3) | 71 (61.7) |
| Ile/Met | 32 (46.4) | 40 (34.8) |
| Met/Met | 3 (4.3) | 4 (3.5) |
| **Allelic 1-df test** [Ile] ↔ [Met] | **P** 0.143 | **OR (95% CI)** 1.44 (0.88 – 2.35) |

Abbreviations: see Supplementary Table 2.

**Supplementary Table 4**

*PNPLA3* genotypes and clinical and laboratory parameters in PBC patients

|  | ***PNPLA3* p.Ile148Met variant** | |  |
| --- | --- | --- | --- |
| **Variables** | **[Ile/Ile]** | **[Ile/Met] + [Met/Met]** | **P** |
| **AST (IU/l)** | 92.8 ± 15.3 | 74.7 ± 5.9 | 0.32 |
| **ALT (IU/l)** | 88.1 ± 13.3 | 82.6 ± 9.3 | 0.75 |
| **AP (IU/l)** | 284.3 ± 22.1 | 335.7 ± 44.9 | 0.27 |
| **γ-GT (IU/l)** | 276.4 ± 32.7 | 329.6 ± 48.7 | 0.35 |
| **Bilirubin (mg/dL)** | 3.5 ± 0.7 | 2.5 ± 0.5 | 0.30 |

Abbreviations: AST, aspartate aminotransferase; ALT, alanine aminotransferase; AP, alkaline phosphatase; γ-GT, γ-glutamyl transpeptidase; Ile, isoleucine; Met, methionine; *PNPLA3*, adiponutrin.

**Supplementary Table 5**

Distribution of *PNPLA3* alleles and genotypes in ICP patients and controls

|  | **Count of alleles / genotypes** | |
| --- | --- | --- |
| ***PNPLA3* p.Ile148Met alleles / genotypes** | **ICP (%)  (n = 201)** | **Controls (%) (n = 198)** |
| Ile | 323 (80.3) | 289 (73.0) |
| Met | 79 (19.7) | 107 (27.0) |
| Ile/Ile | 135 (67.2) | 107 (54.0) |
| Ile/Met | 53 (26.3) | 75 (37.9) |
| Met/Met | 13 (6.5) | 16 (8.1) |
| **Allelic 1-df test** [Ile] ↔ [Met] | **P** 0.013 | **OR (95% CI)** 1.51 (1.09 – 2.11) |

Abbreviations: see Supplementary Table 2. ICP, intrahepatic cholestasis of pregnancy.

**Supplementary Figure 1**

**
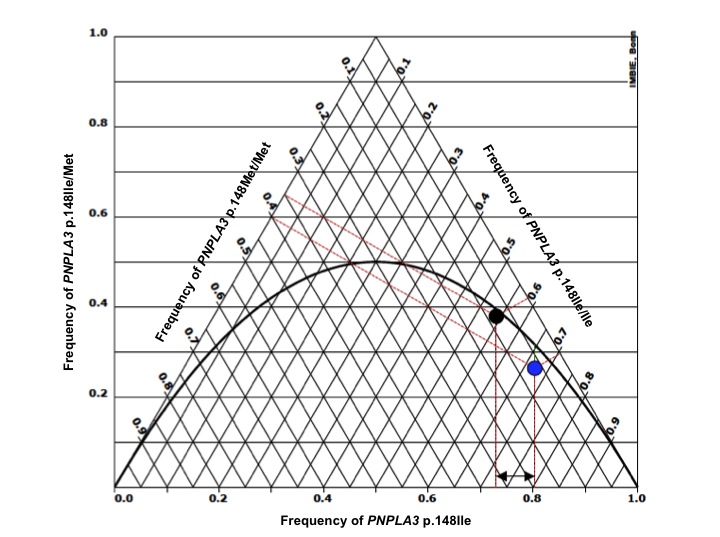
**

**Legend to Supplementary Figure 1**

Intrahepatic cholestasis of pregnancy is associated with the *PNPLA3* allele p.148Ile.

De-Finetti diagram with Hardy-Weinberg parabola for the *PNPLA3* p.Ile148Met polymorphism. The diagram illustrates genotype and allele frequencies in cases (females with ICP; blue dot) and sex-matched controls (black dot) without history of ICP. In this triangular diagram, the frequencies of homozygous carriers of the *PNPLA3* alleles p.148Met and p.148Ile are depicted on the left and right diagonal axes, respectively, whereas the frequencies of heterozygous individuals are plotted on the vertical axis on the left. The frequencies of the *PNPLA3* alleles given at the intersection between the vertical dotted lines and the bottom perpendicular. Genotype frequencies in controls, but not in cases, plot on the diagram’s parabola, which represents all distributions consistent with HWE. ICP cases deviate significantly (P = 0.01) from HWE. The distinct intersections of the red vertical doted lines with the horizontal axis at the bottom illustrate that the frequency of the *PNPLA3* allele p.148Ile is higher in ICP patients as compared to controls.
